# Molecular Characterization of the Gastrula in the Turtle *Emys orbicularis*: An Evolutionary Perspective on Gastrulation

**DOI:** 10.1371/journal.pone.0002676

**Published:** 2008-07-16

**Authors:** Marion Coolen, Delphine Nicolle, Jean-Louis Plouhinec, Aurélie Gombault, Tatjana Sauka-Spengler, Arnaud Menuet, Claude Pieau, Sylvie Mazan

**Affiliations:** 1 Développement et Evolution des vertébrés, UMR 6218, CNRS et Université d'Orléans, Orleans, France; 2 Institut Jacques Monod, UMR7592, CNRS et Université Pierre et Marie Curie-Paris6 and Paris7, Paris, France; University of Maryland, United States of America

## Abstract

Due to the presence of a blastopore as in amphibians, the turtle has been suggested to exemplify a transition form from an amphibian- to an avian-type gastrulation pattern. In order to test this hypothesis and gain insight into the emergence of the unique characteristics of amniotes during gastrulation, we have performed the first molecular characterization of the gastrula in a reptile, the turtle *Emys orbicularis*. The study of *Brachyury*, *Lim1*, *Otx2* and *Otx5* expression patterns points to a highly conserved dynamic of expression with amniote model organisms and makes it possible to identify the site of mesoderm internalization, which is a long-standing issue in reptiles. Analysis of *Brachyury* expression also highlights the presence of two distinct phases, less easily recognizable in model organisms and respectively characterized by an early ring-shaped and a later bilateral symmetrical territory. Systematic comparisons with tetrapod model organisms lead to new insights into the relationships of the blastopore/blastoporal plate system shared by all reptiles, with the blastopore of amphibians and the primitive streak of birds and mammals. The biphasic *Brachyury* expression pattern is also consistent with recent models of emergence of bilateral symmetry, which raises the question of its evolutionary significance.

## Introduction

Analyses focused on a very limited number of model organisms have led to major advances in our understanding of the molecular mechanisms controlling key developmental processes. These studies have deeply impacted our understanding of the unity of metazoans, by showing that as diverse organisms as the mouse, *Drosophila* and even *Hydra* use a relatively small set of related regulatory modules, repeatedly co-opted and adapted to different cellular contexts, to build their body plan. At the microevolutionary scale, they have also paved the way for accurate identifications of genetic modifications responsible for behavioral or morphological diversifications, thus enlightening the genetic architecture underlying these evolutionary processes [1], [2]. However, at the macroevolutionary scale, attempts to reconstruct an evolutionary pathway through comparisons between selected, often distantly related, model organisms or through analyses of mutant phenotypes interpreted as atavisms, often remain hazardous. The major obstacle to these approaches is that each model organism has diverged from ancestral patterns by an accumulation of taxa- or even species-specific changes. Developmental genetics, which mainly focus on phenotypes associated to a very limited number of often dramatic genetic changes such as gene inactivation or ectopic mis-expressions, are unlikely to reconstruct this succession of events. Such a problem is not easily resolved in the absence of extant transition forms but one way to alleviate the difficulty can be to first assess the generality of the mechanisms characterized in a given model organism within a taxon of relatively closely related species. Such comparisons at moderate evolutionary scale must help to infer the ancestral state of a given taxon, which can then be used for comparisons with more distantly related species.

**Figure 1:**
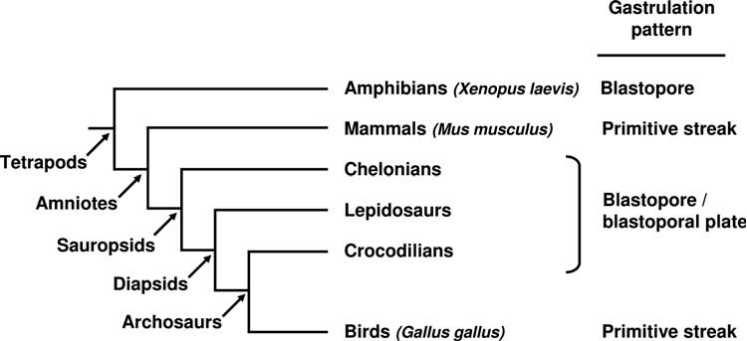
Gastrulation patterns of the major tetrapod groups. The phylogenetic relationships between the major amniote taxa are taken from [45]. Model species are indicated in italics. The mode of gastrulation in each group is shown on the right, as reviewed in [9].

We have used this strategy in order to gain insight into the emergence of the amniote-like gastrulation pattern and better understand its link with the gastrulation patterns observed in amphibians, the sister-group of amniotes. Our current knowledge of this process in amniotes mainly relies on studies conducted in two model organisms, the mouse and the chick. During gastrulation, these two species share a number of unique features, never found in amphibians, despite the substantial variations observed in this taxon [3]–[5]. An obvious difference is that in avians as in mammals, mesendoderm internalization takes place by ingression through an elongated posterior structure, the primitive streak, while in most amphibians it involves involution cell movements at the level of a round-shaped blastopore [6]. Another difference is the presence of an extraembryonic cell population (extra-embryonic ectoderm in the mouse and area opaca in the chick), which encircles embryonic territories and has no equivalent in amphibians. Most studies aimed at understanding the relationships between amniote- and amphibian-type gastrulation patterns have therefore been focussed on the emergence of these characteristics [7], [8]. However, while the latter is shared by all amniotes, the presence of a primitive streak is actually restricted to mammals and avians. In all other species including turtles, lepidosaurs and crocodiles, the closest relatives of birds, no such elongated structure is observed. Instead, gastrulation involves the formation of a blastopore and adjacent marked thickening termed the blastoporal plate, both located close to the posterior part of the area pellucida (Fig. 1) [9]. Together with the presence of a blastopore in amphibians, the phylogenetic distribution of the blastopore/blastoporal plate pattern among amniotes (Fig. 1), has been taken as evidence for its ancestral character in the taxon, suggesting that the avian and mammalian primitive streaks may represent independent modifications of this system [9]. A better understanding of the relationships between these structures is important to delineate the amniote ancestral state and the specificities of their model organisms. However, no molecular characterization of gastrulation has been thus far reported in a reptile, which makes accurate comparisons difficult. In order to better understand the relationships between the primitive streak of birds and mammals, blastopore of amphibians and blastopore/blastoporal plate system of reptiles, we have analyzed the expression pattern of four regional markers of gastrulation in a turtle, *Emys orbicularis*. Analysis has been more particularly focussed on regional markers of internalizing mesoderm in amniote model organisms, such as *Brachyury*, often considered as a general primitive streak marker, *Lim1*, restricted to the anterior primitive streak throughout gastrulation, and *Otx2*, which displays a dynamic expression pattern in the early anterior primitive streak, organizer and its derivatives, in addition to a prominent later anterior neuroectoderm expression. These data lead to a reassessment of current models of the emergence of amniote characteristics during gastrulation and highlight an unexpected parallel with recent hypotheses on the origin of the vertebrate embryonic axes.

## Materials and Methods

### Turtle embryos and staging

Permission from the French Ministry of Ecology was given to C. P. to capture a maximum of 60 gravid females, collect eggs and incubate them in controlled laboratory conditions, one proportion of them being kept for scientific research and the rest released either on the site of capture or placed in a breeding unit (Ferme aux crocodiles, Pierrelatte, France) for a program of reintroduction of endangered species (arrêtés n° 2003-E-2504DDAF/457 and n°2004-E-1161 DDAF/185). This permission was given following advice of the Conseil National de la Protection de la Nature. Adult animals were released on their site of capture following spawning. A total of 150 eggs were used for these analyses. Eggs were incubated at 30°C and dissected at gastrulation or early neurulation stages. Experimental procedures on early embryos comply with institutional regulations (code rural article R 214-90). Stages were determined following the criteria described in *Chelydra serpentina* and *Testudo hermanni* [10], [11] from stage 2 onwards. At earlier stages (stages 0–1), we carried out systematic histological analyses in order to better understand the changes in morphology at the earliest available stages in *E. orbicularis*.

### Nomenclature

#### Posterior-ventral, anterior-dorsal

The orientation of the blastoporal canal changes between stages 0 and 1, being converted from a vertical elongated cavity to an almost horizontal one. For this reason, we refer to the blastopore rims as posterior or anterior at the earliest stage studied (stage 0a), and ventral or dorsal starting from stage 0b. This nomenclature thus refers to the future polarities of the embryo proper when it becomes visible.

#### Reptilian

Even though reptiles (which comprise chelonians, lepidosaurs and crocodilians) form a paraphyletic group, we refer to their mode of gastrulation as “reptilian” for convenience purposes. The “reptilian” gastrulation pattern, characterized by the presence of a posterior blastopore and blastoporal plate, is very similar in chelonians, lepidosaurs and crocodilians.

### Probe amplification

*Otx2*, *Otx5, Lim1* and *Brachyury* probes were obtained by degenerate PCR starting from *E. orbicularis* genomic DNA or stage 1 cDNA as described previously [12]–[14]. The degenerate primers used and sequences of the resulting amplified products are shown in Supplementary Figure S1. Their identity was confirmed by systematic reverse Blast analyses against Genbank.

### ISH and sectioning

Turtle embryos were obtained as described in [15], fixed overnight in paraformaldehyde 4% in PBS (PFA 4%), and stored dehydrated in methanol 100%. Whole-mount *in situ* hybridizations were conducted using *in vitro* synthesized digoxigenin-labeled antisense RNAs, after the protocol described in [12] with the following adaptation. The proteinase K treatment was carried out at a concentration of 10 µg/ml for 10 minutes at room temperature from stages 0 to 4 and extended to 20 minutes at later stages. Histological analyses were systematically carried out following *in situ* hybridizations, using cryostat sections as described previously [12].

## Results

### Morphological aspects of gastrulation in the turtle *Emys orbicularis*

**Figure 2:**
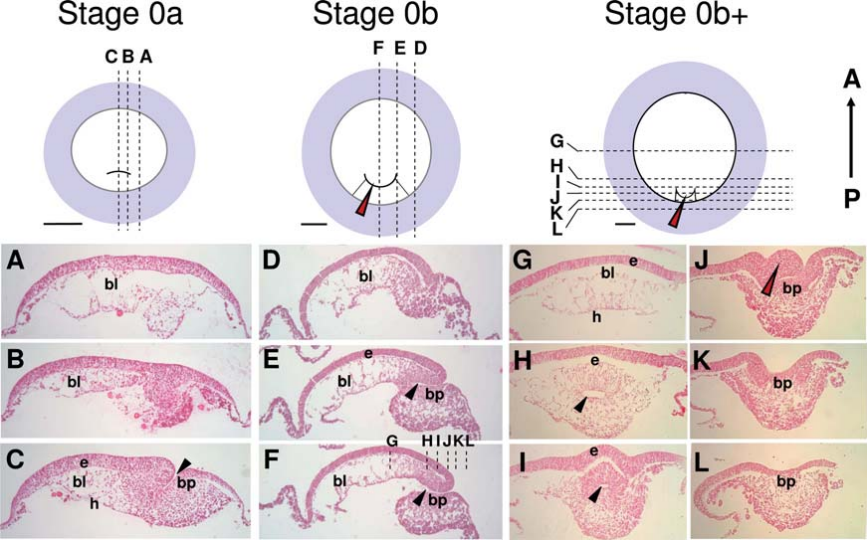
Histological characteristics of stage 0 *E. orbicularis* embryos. Schematic dorsal views of turtle embryos at stages 0a to late 0b (0b+) are shown in the upper line, with the area opaca in blue and the area pellucida in white. The level and plane of sections shown in A–L are indicated by dotted lines on the schematic views. The approximate location of the sections shown in G–L are also shown on a tranverse section (F) of a slightly younger embryo to help interpretation of the histologies. Red arrowheads in J points to a characteristic protrusion of the dorsal lip, which starts to form at stage 0b and appears as a narrow extension between the lateral lips at late stage 0b. Section J is a tangential section of this protrusion. Black arrowheads indicate the blastoporal cavity or canal. A–C, D–F : sagittal sections of respectively stage 0a and 0b embryos; G–L : transverse sections of a late stage 0b embryo. bl, blastocoel; h, hypoblast; bp, blastoporal plate; e, epiblast. Scale bar : 500 µm.

**Figure 3:**
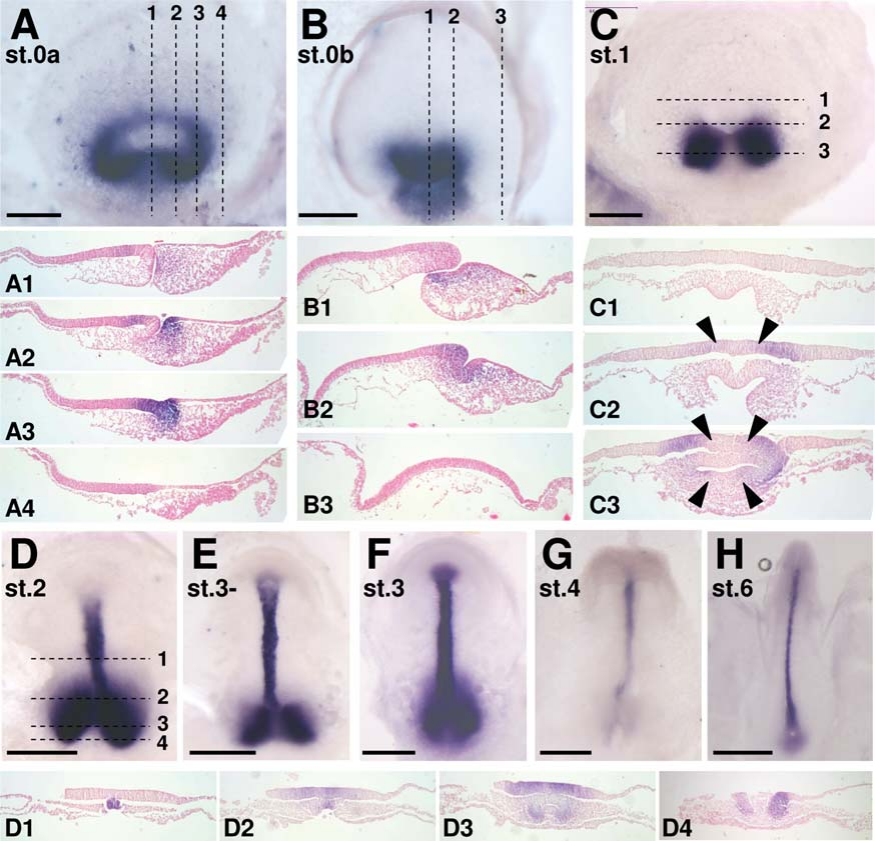
*Brachyury* expression during gastrulation in *E. orbicularis.* A–C, D–H: whole mount views of *E. orbicularis* embryos after in situ hybridization with the *Brachyury* probe (A–B, D–H: dorsal views; C: ventral view). A1-4, B1-3, C1-3, D1-4: sections of embryos shown in A, B, C, D respectively. The planes of sections are indicated by dotted lines on the whole-mount view of each embryo. The signal is ring-shaped at stages 0a–0b (A, B) but shows a clear bilateral symmetry at stage 1, with a discontinuity at medial levels. The negative territories observed in the midline of both the dorsal lip and blastoporal canal floor are delimited by black arrows. Scale bar: 500 µm.

**Figure 4:**
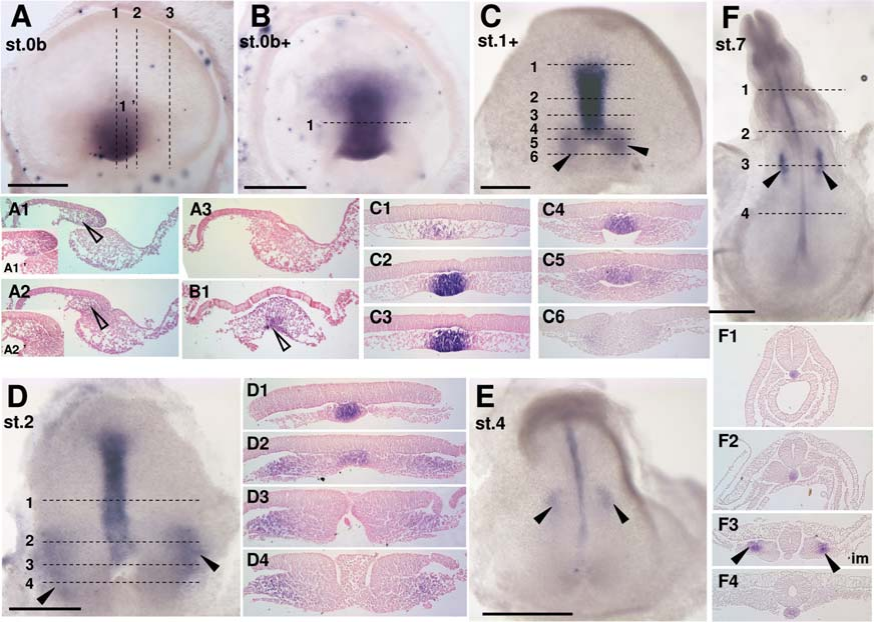
*Lim1* expression during gastrulation in *E. orbicularis.* A–F: dorsal views of *E. orbicularis* embryos after in situ hybridization using the *Lim1* probe. A1-3, B1, C1-6, D1-4, F1-4: sections of the embryos shown in A, B, C, D, F respectively. Higher magnifications of A1 and A2 at the dorsal lip level are shown in A1' and A2'. Opened arrowheads in A1-2 and B1 point to the *Lim1* positive mesenchymal cell population. The planes of sections are indicated by dotted lines on the whole-mount view of each embryo. Black arrowheads in C, D, E, F, F3 point to the prospective intermediate mesoderm expressing *Lim1* starting from stage 1+. Scale bar: 500 µm.

Developmental tables have been published for two turtle species, *Chelydra serpentina* [10] and *Testudo hermanni boettgeri* [11] with congruent stage definitions. These descriptions, mainly based on external morphological changes, were also found to largely apply to *E. orbicularis*. However, they do not address the histological changes observed between stage 0 and stage 1, respectively defined by the appearance of the blastopore and cephalic enlargement. We therefore performed a detailed histological description of *Emys orbicularis* embryos prior to stage 1. As previously described [reviewed in 9], the blastopore appears shortly after oviposition, as a transverse fold in the posterior third of the blastoderm (dorsal view in Fig. 2). As visible in sections (Fig. 2A) an elongated transverse cavity extends anteriorly and ventrally towards the yolk. Three cell populations are observed at this stage, a superficial epithelium, markedly thickened in the major part of the blastoderm and consisting of a single-cell layer peripherally, a thin lower epithelial cell sheet in direct contact with the yolk, and intermediate mesenchymal cells (Fig. 2A–C). Following anatomists, we refer to the superficial and lower cell layers as epiblast and hypoblast respectively. The mesenchymal cells are partitioned into two distinct populations, one that appears dispersed in the blastocoel cavity in the anterior moiety of the blastoderm, and the other more compact, around the elongating blastopore and posterior to it, under the epithelial layer of the blastoporal plate. The absence of a bilayered organization around the blastopore rims suggests that delamination cell movements from the epiblast prevail at this stage. We refer to this stage as stage 0a, based on two criteria, an external one, the shape of the dorsal blastopore lip, which consistently appeared horizontal in these early embryos, and an histological one, the absence of a bilayered organization indicative of involution cell movements at this level. The ventral extension of the blastopore cavity could be more or less pronounced in embryos displaying these characteristics, a fusion between the blastocoel walls and hypoblast taking place at late stage 0a. At stage 0b, the dorsal lip of the blastopore changes shape, with a slight posterior extension. The blastoporal cavity now shows a marked anterior to posterior orientation (Fig. 2E–F) and a bilayered organisation, indicative of epiblast involution movements, becomes obvious at the anterior and lateral rims of the blastopore (Fig. 2D–F). In contrast, no indication of involution could be observed at the posterior/ventral lip level, histological views rather suggesting a global delamination (Fig. 2E–F). These broad characteristics are maintained at late stage 0b (0b+ in Fig. 2G–L), with the following modifications. First, the previously slit-shaped blastopore opening narrows, thus forming the blastoporal canal (Fig. 2J). The posterior extension of the dorsal lip, which started to form at stage 0b, becomes more pronounced and now markedly protrudes between the lateral lips (red arrowhead in Fig. 2J). This structure completely regresses at stage 1, characterized by the appearance of two prominent bilateral ventral bulges [10, 11 ; see Fig. 3 and 4, this study].

### *Brachyury* expression pattern highlights distinct sites of mesoderm internalization at the onset of gastrulation and during subsequent stages

Together with histological analyses, vital dye labeling experiments pointed to the blastopore lips and possibly floor as the major sites of mesendoderm internalization [14], [15], [reviewed in 9]. In order to more accurately address this issue, we analyzed expression of *Brachyury*, a general marker of internalizing mesendoderm in chordates [12], [18]–[22]. At the first stage studied (0a, Fig. 3A), the expression domain appears as a continuous ring encircling the blastoporal region, with the highest signal intensity in postero-lateral regions (dorsal view in Fig. 3A). At anterior-most levels, in the midline, the signal is restricted to a narrow band of the epiblast layer, at a distance from the blastopore anterior lip, and excludes both the involuting anterior lip and adjacent delaminating anterior mesenchymal cells (Fig. 3A1–A2). At lateral and postero-lateral levels, it spans the thickened epiblast layer lining the blastoporal canal and adjacent compact mesenchyme of the blastoporal plate (Fig. 3A2–A3). The signal persists posteriorly at medial levels, albeit with a marked decrease in intensity (Fig. 3A1). At stage 0b, expression in the epiblast withdraws from the anterior, midline territory (Fig. 3B1) and concentrates along the lateral lips of the blastopore opening and the deepest parts of the floor of the blastoporal canal (Fig. 3B1–B2). Expression persists in the adjacent mesenchyme, that posteriorly forms a stream of elongated cells extending below the superficial epithelial layer and towards the posterior limit of the blastoderm (Fig. 3B1–B2). At stage 1, the broad characteristics of the labeled territory radically change. Transcripts become restricted to two bilateral sites of expression, comprising the involuting lateral lips, adjacent epiblast and mesenchyme. The signal thus completely disappears from the midline, both from the epiblast involuting at the dorsal rim of the blastoporal canal opening and from the floor of the blastoporal canal and underlying mesenchyme (Fig. 3C, C1–C3). These characteristics of *Brachyury* expression around the blastopore are maintained at later stages with two modifications. The bilateral expression territories previously excluding the dorsal midline now include the dorsal blastopore lip (Fig. 3D, 3D2–D4). In addition, as in all chordates, the forming notochord, which extends anterior to the *Brachyury* positive dorsal-most involution zone, becomes a major expression site (Fig. 3D–H, 3D1).

### *Lim1* expression territory : axial mesendoderm and intermediate mesoderm formation in the turtle

*Lim1* displays highly conserved expression sites during gastrulation in amniotes. Initially located to the hypoblast, transcripts are later found in the anterior part of the primitive streak, axial mesendoderm including prechordal cell populations, and intermediate mesoderm of the urogenital system [23]–[25]. In order to identify homologous cell populations in the turtle, we analyzed *Lim1* expression starting from stage 0b. At stage 0b to 0b+, a prominent signal is observed in the involuting dorsal lip of the blastopore, the mesenchyme and hypoblast layers lying anterior to it (Fig. 4A, 4A1–A3; Fig. 4B, 4B1). At these stages, the signal completely excludes the lateral and ventral blastoporal plate. Expression in the dorsal blastopore lip persists until stage 2 (Fig. 4C, 4C5) and is still visible at later stages, albeit with a fainter signal intensity. The adjacent elongating axial mesendoderm (Fig. 4C2–C4) and the looser mesenchymal cells lying anterior to it are also labeled (Fig. 4C1) and this signal persists at later stages (Fig. 4D, 4D1; Fig. 4E; Fig. 4F, 4F1–F4). An additional expression site along the lateral lips appears at late stage 1 (Fig. 4C, 4C5–C6). Transcripts are detected not only in the involuting epiblast layer but also in cells ingressing laterally from this area. At subsequent stages, *Lim1* expression becomes localized to lateral regions of the mesoderm (Fig. 4D, 4D1–D4; 4E) and is later restricted to the intermediate mesoderm component (stage 4; Fig. 4F3). No neural crest or neuroectoderm signal could be observed at the stages studied and analyses at later stages will be important to assess their conservation in tetrapods.

### Anterior neuroectoderm specification in the turtle: insights from *Otx2* and *Otx5* expression

**Figure 5:**
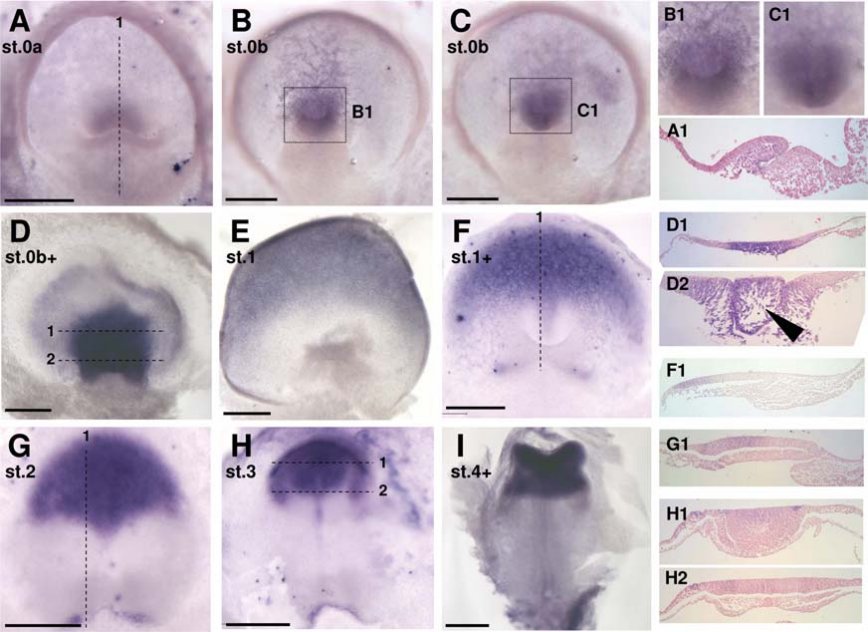
*Otx2* expression during gastrulation in *E. orbicularis.* A–I: whole mount views of *E. orbicularis* embryos after in situ hybridization using the *Otx2* probe (A, C–I: dorsal views; B: ventral view of the same embryo as in C). B1, C1: higher magnification of the region of the blastopore region of the embryo shown in B and C. A1, D1-2, F1, G1, H1-2: sections of hybridized embryos shown in A, D, F, G, H respectively. The planes of sections are indicated by dotted lines on the whole-mount view of each embryo. Black arrowhead in D2 points to the dorsal epiblast extension protruding between the lateral lips of the blastopore. Scale bar: 500 µm.

**Figure 6:**
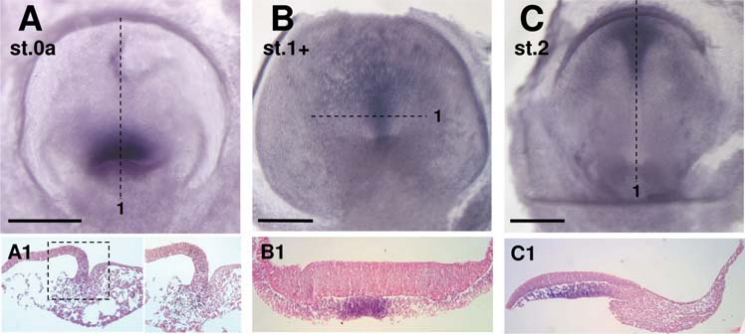
*Otx5* expression during gastrulation in *E. orbicularis.* A–C: whole mount views of *E. orbicularis* embryos after in situ hybridization using the *Otx5* probe (A, C: dorsal views; B: ventral view). A1, B1, C1: sections of hybridized embryos shown in A–C respectively. A1': higher magnification of A1 at the anterior lip level. The planes of sections are indicated by dotted lines on the whole-mount view of each embryo. Scale bar: 500 µm.

In both chick and mouse, early forebrain formation involves a complex succession of inductive events [26], [27]. *Otx2* plays important cell-autonomous and non cell-autonomous roles in this process and shows a highly dynamic expression pattern in the hypoblast, early node and anterior neuroectoderm from pre- to late-streak stages [25], [28]–[33]. We addressed the conservation of these steps in the turtle using this marker along with the paralogous gene *Otx5*. Both genes show very similar expression patterns at stage 0a (Fig. 5A, 6A). At these stages, transcripts are detected in the involuting epiblast layer at the anterior rim of the blastopore and adjacent mesenchyme cell populations, including the roof of the blastoporal canal (Fig. 5A1, 6A1). At stage 0b, *Otx2* expression extends further anteriorly in the lower hypoblast layer, where positive and negative cells are intermingled (Fig. 5B, 5B1). The *Otx2* signal markedly intensifies at late stage 0b (Fig. 5D, 5D1–D2). Expression then becomes prominent in the involuting lateral lips, the dorsal epiblast extension protruding between them, and adjacent ingressing mesenchymal cells. However, it remains undetectable in the ventral rim and floor of the blastopore. Starting from stage 1, *Otx2* and *Otx5* territories become clearly distinct. *Otx2* expression spans a broad 180° crescent shaped sector in the anterior half of the thickened epiblast and underlying mesendoderm (Fig. 5E, 5F, 5F1). The posterior borders of this territory sharpen at subsequent stages. The labeling then becomes prominent in the anterior neuroectoderm and neighbouring presumptive surface ectoderm at neural plate stages and upon neural tube closure (Fig. 5G–I, 5G1, 5H1–H2). In contrast, at stage 1, *Otx5* transcripts are restricted to an anterior medial 30° sector, originating from the dorsal lip of the blastopore (Fig. 6B). The signal is present in the mesendoderm, adjacent loose mesenchyme layer and hypoblast but excludes the *Otx2* positive ectoderm (Fig. 6B1). The labeled area persists at stage 2 but is displaced to the anterior third of the elongating axis (Fig. 6C, 6C1).

## Discussion

Classic anatomical studies have left a number of unanswered questions on the relationships between turtles, amphibian and amniote model organisms during gastrulation. The molecular characterization leads to a more accurate picture of similarities and differences, thus providing a basis to reassess current models of transition from amphibian- to amniote-gastrulation patterns.

### A highly conserved dynamic of *Otx* expression between the turtle and the chick : a basis for stage comparisons

*Otx2* plays a highly conserved, central role in early head formation in all chordates. Functional dissections, particularly those conducted in amniotes, have shown that the gene was actually involved in at least three distinct processes during gastrulation, all related to different aspects of head formation. It is thus successively involved in the repression of posteriorising signals by the hypoblast [28], [30], the stable specification of anterior neuroectoderm by the early node and its derivatives (prechordal mesendoderm), and, at early neural plate stages, in the cell-autonomous maintenance of anterior neuroectoderm cell fate [31], [32]. In line with these roles, *Otx2* displays a highly dynamic expression profile between the earliest stages of streak formation to node regression, which has been best documented in the chick [25], [29]. Initially restricted to the hypoblast (stages XIII-XIV), transcripts also become detectable in the anterior part of the forming primitive streak at stage HH2 [25], [29]. When the streak reaches its maximal extension (stages 3d, [25]), the signal persists at the node level but completely withdraws from the hypoblast. Finally, by stage 4, it becomes undetectable in the regressing primitive streak and confined to the anterior neuroectoderm and underlying prechordal mesendoderm [25]. The posterior boundary of the anterior neuroectoderm territory sharpen at subsequent head process and head fold stages (HH5-6). The dynamic of *Otx2* expression observed in the turtle shows strong similarities with this profile. The initial expression in the thin hypoblast layer and dorsal blastopore lip at stages 0a-0b is thus reminiscent of the stage HH2 expression observed in the chick. Similarly, at late stage 0b, the disappearance of the hypoblast signal concomitant with a marked increase at the dorsal lip, recalls the signal observed in the chick anterior streak at late stage HH3. Finally, as in the chick (stages HH5-6), expression in the turtle then completely leaves the site of mesendoderm internalisation (blastopore lips in this case) and becomes prominent in the anterior neuroectoderm and underlying mesendoderm with sharp posterior boundaries (stages 2 to 3). Taken together, these similarities suggest a strong conservation of essential specification processes between the chick and the turtle and provide a molecular basis for stage equivalencies, as summarized in Table 1. They also point to the stage 0 dorsal lip as the homologue of the anterior part of the early primitive streak. The hypoblast and dorsal lip expressions of *Lim1* in the turtle, which can similarly be related to early hypoblast and later anterior streak expressions in the chick, are consistent with both conclusions. Finally, it should be noted that the paralogous *Otx* gene *Otx5* is also transcribed in the dorsal blastopore lip and the prechordal mesendoderm possibly derived from it during gastrulation in the turtle, suggesting a possible functional redundancy with *Otx2*. Such a coexpression has been reported in *Xenopus* but has not been documented in the mouse or the chick, suggesting that the turtle may reflect the amniote ancestral state, lost in amniote model organisms.

**Table 1:** Comparison of gastrulation and early neurulation stages between the turtle and the chick.

*E. orbicularis*	0a-0b	1	2	3
***G. gallus***	HH2-HH3	HH4	HH5	HH6

Stage correspondances at turtle stage 0 to 2 were mainly based on *Otx2* expression pattern, which appears highly dynamic and very similar in the turtle and in the chick (see Dicussion). Additional morphological criteria were used at turtle stages 2 and 3, respectively characterized by the presence of the notochord in its anterior-most parts (head process : stage HH5 in the chick and 2 in the turtle), and the elevation of the anterior neural folds (head fold : stage HH6 in the chick and 3 in the turtle).

### Relationships between blastopore and primitive streak gastrulation patterns in amniotes: towards the amniote ancestral state

Attempts to identify primitive streak equivalents in the turtle on the basis of embryonic morphologies have led to controversial interpretations. Based on histological analyses in *Caretta caretta*, Mitsukuri suggested the presence of a primitive streak-like structure at the level of the late blastoporal plate [34], [reviewed in 9]. However, no such structure was reported in other turtles. Cell labeling experiments using vital dye staining in *Clemmys* led to another hypothesis, since they pointed to the blastopore lips as the main sites of mesendoderm internalization [16], [17]. The molecular characterization provides new insights into this issue in *E. orbicularis*. First, whatever the stage analyzed, we found no indication of an elongated, primitive streak-like structure at the blastoporal plate level. The internalization of *Brachyury* positive mesendoderm at this level appeared restricted to a narrow transverse territory located deep inside the blastoporal canal, and only observed prior to stage 1. Similarly, at later stages, the *Brachyury* positive territory was found to completely exclude the blastoporal plate, since it became restricted to the dorsal and lateral rims of the blastopore, extending into the adjacent anterior notochord. These results appear fully consistent with the results of pioneering vital dye cell labeling experiments, performed more than 50 years ago [17]; [reviewed in 9].

Another unexpected outcome of our analysis is that the *Brachyury* expression pattern shifts from a ring-like to a horse shoe-shaped domain by stage 1, which according to the stage equivalencies proposed above, may correspond to maximal streak extension in the chick. This highly dynamic biphasic expression pattern seems to substantially differs from the apparently stable expression of *Brachyury* along the primitive streak described in amniote model organisms. However, despite the absence of overt morphological changes of the primitive streak during gastrulation, a very similar transition may also take place in birds and mammals, between streak elongation and regression stages (respectively HH2-HH4 and HH4-HH6, corresponding to stages 0–1 and 1–3 in the turtle). In support of this possibility, high resolution analyses of cell movements recently reported in the chick have shown that, shortly after stage XII, cell converge not only to the “future primitive streak axis” (“vertical” axis), aligned along the future antero-posterior polarity of the embryo itself, but also more unexpectedly to a transverse (“horizontal”) territory located near the edge of the area pellucida and described “as an arc parallel to the posterior marginal zone” [8]. This latter territory is reminiscent of the early *Brachyury* positive transverse domain observed at the floor of the blastoporal canal in the turtle. It could thus be a remnant of the posterior rim of the ancestral reptilian blastopore, whereas the other, “vertical” site of cell convergence reported in the chick could be evolutionarily related to its dorsal and lateral lips. Similarly, comparisons between the turtle and the rabbit, which as the chick and actually most mammals, develops as a flat blastoderm thus yielding excellent spatial resolutions of expression patterns, point to another noticeable similarity. In the latter, the anterior part of the primitive streak is negative for *Brachyury* expression at stages 3–4, prior to the onset of node regression and head process formation, except for a transient, discontinuous node expression phase [35]. We could not detect a clear equivalent of this transient node expression, which may have escaped detection in the turtle. In contrast, the exclusion of *Brachyury* transcripts from anterior streak in the rabbit is clearly reminiscent of the absence of expression observed in the turtle at the *Lim1* and *Otx* expressing dorsal blastopore lip until stage 1, just prior to the onset of anterior notochord elongation, which only becomes clearly visible at stage 2. This early internalized *Lim1* and *Otx* expressing mesendoderm cell population may thus correspond to prechordal mesendoderm, negative for *Brachyury* in all vertebrates [12]. Cell fate analyses will be essential to validate this interpretation. Taken together, these data suggest that the two phases of *Brachyury* expression, which appear conspicuous in the turtle, may be general among amniotes.

### Comparisons between amphibian- and amniote-like gastrulation patterns : insights from the turtle

**Figure 7:**
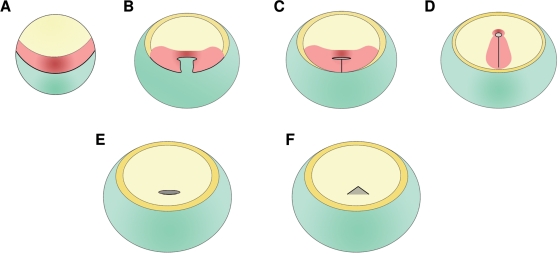
Comparison of the turtle gastrula (E,F) with hypothetical ancestral forms (C, D) accounting for the transition between amphibian- and avian-like gastrulation patterns. The first line depicts the evolutionary scenario proposed by Arendt and Nübler-Jung (1999) to account for the transition between an amphibian-like (A) and an avian-like (D) gastrulation pattern. Two successive transition forms have been hypothesized in this model. The first one (B), proposed to be ancestral to amniotes, is characterized by a posterior restriction of the site of mesoderm internalization (black line). The following one (C), supposedly tortoise-like and ancestral to reptiles, is characterized by a fusion of the bilateral sites of mesoderm internalization, along a structure related to the blastoporal plate. The turtle gastrulation mode (symbolized in E at stages 0a-0b and in F starting from stage 1) as assessed by this study is consistent with the conclusion that an increase in yolk cell mass may have led to the posterior restriction of the site of mesoderm internalization. However, the turtle differs from the hypothetical intermediate shown in C in that we have no indication of mesoderm internalization along a possible remnant of the line of posterior fusion, running along the dorsal part of the blastoporal plate and abutting the blastoderm posterior margin as in C. At stages 0, the site of mesendoderm internalization is ring-shaped and runs across the blastoporal plate (E), while it is restricted to the dorsal and lateral blastopore lips starting from stage 1 (F). Whatever the stage, it is located *inside* the blastoderm, at a distance from the posterior margin, unlike in the hypothetical ancestor shown in C. Green: vegetal hemisphere, light yellow: ectoderm in A–D or epiblast in E–F; orange: extraembryonic ectoderm; red, presumptive mesendoderm in A–D; this cell population is not shown in E–F, since cell movements and fate maps were not addressed in the turtle.

*Xenopus*- and avian-like gastrulation patterns differ by a number of distinctive features, which in the latter are likely to correspond to derived characteristics, such as the segregation and radial expansion of territories that do not contribute to the embryo proper, as well as a posterior restriction and characteristic elongation of the site of mesendoderm internalization. More than ten years ago, Arendt and Nübler-Jung proposed a hypothetical sequence of progressive modifications to account for the emergence of these characteristics [7]. This model relied on three crucial steps, (1) a progressive withdrawal of the presumptive mesoderm from ventral territories, (2) a fusion of the resulting posterior wings of internalizing mesoderm, at the level of a structure proposed to be related to the blastoporal plate of modern turtles, and (3) an elongation of this structure thus converted into an avian-type primitive streak (Fig. 7A–D). The reconstruction of the amniote ancestral state, as inferred by comparisons between the turtle and either the chick or amphibians, confirms several key features of this model. It first adds support to the predicted homology between the anterior rim of the turtle blastopore and the *Xenopus* dorsal lip (Fig. 7A and 7E). The involution cell movement, likely corresponding to an ancestral character retained by turtles, thus prevails at this level in both species. Similarly, these territories share expression of organizer markers. In particular, *Lim1*, as well as both *Otx2* and *Otx5* are specifically expressed at the dorsal lip of the blastopore or Spemann organizer in *Xenopus*, throughout gastrulation for the former and during early stages (stage 10–10.5) for the latter two [36], [37], [38], [39], exactly as observed in the turtle. A very similar dynamic of *Brachyury* expression, initially located at a short distance anterior to the dorsal lip, is also observed in the turtle as in amphibians [40]. As also predicted by the model, the characterization of the turtle gastrula is consistent with a posterior restriction of presumptive ventral mesoderm and its complete withdrawal from the blastoderm margin, which as in the chick, never expresses *Brachyury*. While such a restriction of mesendoderm internalization to one side of the embryo, also observed in salamanders, gymnophionans and to a large extent selacians [5], [12], may be a general evolutionary tendency, repeatedly associated with an increase of the yolk mass, its complete exclusion from the blastoderm margin remains an amniote specificity, shared by the turtle, as observed in [9]. Finally, the occurrence of the last step of the model, hypothesized to account for the conversion of the reptilian blastopore into the elongated avian primitive streak, has also recently gained support from analyses of the genetic mechanisms controlling streak extension at pre-gastrulation stages. This morphogenetic process has been shown to rely on the Wnt-PCP pathway, suggesting that the recruitment of additional medial cell intercalation movements may have been involved in the emergence of the primitive streak during evolution [8]. The characterization of the turtle gastrulation pattern fully supports this conclusion and since no such elongated structure is observed in the turtle, suggests that this modification may have taken place among amniotes rather than prior to their emergence.

Despite these congruencies, the turtle gastrula does not resemble the ancestral, supposedly tortoise-like form, depicted by Arendt and Nübler-Jung in their model (compare Fig. 7C and 7E). We thus find no evidence of an elongated site of mesoderm internalisation abutting the posterior blastoderm margin. This does not preclude the possibility that the transition from an amphibian-to an amniote-like gastrulation mode may have involved the proposed transition form but our work shows that extant turtles do not exemplify this hypothetical ancestral state. Other evolutionary scenarios, involving for instance an earlier radialization of the extra-embryonic ectoderm and concomitant re-localization of mesoderm inducing factors towards the center of the blastoderm remain equally opened.

### An ancient origin for the gnathostome gastrulation pattern?

Theoretical analyses combining mathematical modeling and comparisons at large evolutionary scale, between diploblasts and bilaterians, have led to the suggestion that the construction of the vertebrate body axis may involve the combined action of two signaling centers, or organizers, of very different evolutionary origins [41], [42]. According to this model, the first one, located at the blastopore margin and involved in brain antero-posterior patterning, may be derived from an ancient organizer, already present in the last common ancestor of diploblasts and bilaterians. In line with this hypothesis, the blastoporal region acts as a genuine organizer in *Hydra* or *Nematostella vectensis*, and the pattern which it generates along the unique axis of the body column appears related to the antero-posterior pattern of the brain of extant vertebrates [43], [44]. The second signaling system, of more recent origin, corresponds to the Spemann organizer homologue, proposed to primarily control dorso-ventral polarity during subsequent axis elongation [41], [42]. The action of this second organizer may have played a crucial part in the emergence of the vertebrate body plan, by converting a radially to a bilaterally symmetrical animal form. It is intriguing to note that the gastrulation pattern observed in *Emys orbicularis* is strikingly similar to the prototypical vertebrate pattern hypothesized in this model. In the turtle, *Brachyury* expression thus shifts from a largely radial symmetrical ring to a territory exhibiting a clear bilateral symmetry. A similar shift between a radial and a bilaterally symmetrical expression territory is also observed in the dogfish, despite the extensive morphological divergence and evolutionary distance between the turtle and the shark [12], [13]. Detailed analyses of the signaling systems controlling antero-posterior and dorso-ventral patterning in vertebrates combined with systematic comparative analyses aimed at reconstructing the ancestral state of the major metazoan taxa, will be essential to assess the evolutionary significance of this observation. Taking advantage of the characteristics of non-model organisms such as the turtle, which in some cases lend themselves to more straightforward interpretations than model organisms, could be important in such comparative approaches.

## Supporting Information

Figure S1 Partial E. orbicularis Lim1, Brachyury, Otx2 and Otx5 sequences used as probes in this study.
(0.03 MB DOC)
