# Supplementary material for: Molecular Characterization of the Gastrula in the Turtle Emys orbicularis: An Evolutionary Perspective on Gastrulation
Source: PLoS One. 2008 Jul 16;3(7):e2676. doi: 10.1371/journal.pone.0002676 (PMC2442194; doi:10.1371/journal.pone.0002676)
Supplement: Figure S1 — Partial E. orbicularis Lim1, Brachyury, Otx2 and Otx5 sequences used as probes in this study. (0.03 MB DOC) [file pone.0002676.s001.doc]

# Eo Lim1: Accession number EU741246

V Q C C E C K C N L T E K C F S R E G K L Y C K N D F F R C F G T K C A G C A Q G I S P S N L V

GTNCARTGYTGYGAATGTAAATGCAATTTGACAGAGAAATGCTTTTCCAGAGAAGGCAAGCTTTACTGCAAAAACGACTTCTTTCGGTGTTTTGGGACCAAGTGTGCTGGCTGTGCCCAGGGGATCTCCCCCAGCAACTTGGTC

ATTTACGTTAAACTGTCTCTTTACGAAAAGGTCTCTTCCGTTCGAAATGACGTTTTTGCTGAAGAAAGCCACAAAACCCTGGTTCACACGACCGACACGGGTCCCCTAGAGGGGGTCGTTGAACCAG

R R A R S K V F H L N C F T C M M C N K Q L S T G E E L Y I I D E N K F V C K E D Y L N N S N T

AGGAGGGCGAGAAGCAAAGTGTTCCACTTGAACTGTTTTACTTGTATGATGTGTAACAAACAGCTCTCCACCGGCGAGGAGCTCTATATTATCGACGAGAACAAGTTTGTCTGCAAAGAAGATTACCTAAATAACAGCAATACT

TCCTCCCGCTCTTCGTTTCACAAGGTGAACTTGACAAAATGAACATACTACACATTGTTTGTCGAGAGGTGGCCGCTCCTCGAGATATAATAGCTGCTCTTGTTCAAACAGACGTTTCTTCTAATGGATTTATTGTCGTTATGA

A K E N S L H S A T T G S D P S L S P D S Q D P S Q D D A K D S E S A N V S D K E A G N N E N D

GCCAAAGAGAACAGCCTGCACTCAGCCACAACCGGCAGTGACCCCAGTCTGTCTCCGGATTCCCAAGACCCTTCCCAAGACGACGCGAAAGACTCGGAGAGCGCAAACGTGTCAGACAAGGAAGCTGGCAACAACGAGAATGAT

CGGTTTCTCTTGTCGGACGTGAGTCGGTGTTGGCCGTCACTGGGGTCAGACAGAGGCCTAAGGGTTCTGGGAAGGGTTCTGCTGCGCTTTCTGAGCCTCTCGCGTTTGCACAGTCTGTTCCTTCGACCGTTGTTGCTCTTACTA

D Q N L G A K R R G P R T T I K A K Q L E T L K A A F A A T P K P T R H I R E Q L A Q E T G L N

GATCAGAACCTGGGGGCCAAGAGGCGGGGTCCCCGCACCACTATCAAAGCCAAACAACTTGAAACTCTGAAAGCAGCCTTCGCAGCGACCCCCAAACCCACCAGGCACATCAGGGAACAGCTGGCCCAAGAAACCGGGCTCAAC

CTAGTCTTGGACCCCCGGTTCTCCGCCCCAGGGGCGTGGTGATAGTTTCGGTTTGTTGAACTTTGAGACTTTCGTCGGAAGCGTCGCTGGGGGTTTGGGTGGTCCGTGTAGTCCCTTGTCGACCGGGTTCTTTGGCCCGAGTTG

M R V I Q V W F Q N R R S K E R R M K Q L S A L G A R R H A F F R S P R R M R P L V D R L E P G

ATGCGAGTCATACAGGTGTGGTTCCAGAACCGGCGCTCCAAGGAGCGGCGCATGAAGCAGCTAAGCGCGCTGGGGGCCCGCCGGCACGCCTTCTTCCGCAGCCCCCGCAGGATGAGGCCGCTGGTGGACCGGCTGGAGCCCGGG

TACGCTCAGTATGTCCACACCAAGGTCTTGGCCGCGAGGTTCCTCGCCGCGTACTTCGTCGATTCGCGCGACCCCCGGGCGGCCGTGCGGAAGAAGGCGTCGGGGGCGTCCTACTCCGGCGACCACCTGGCCGACCTCGGGCCC

E L I P N G P F S F Y G D Y Q S E Y Y G P G S N Y D F F P Q G P G

GAGCTCATCCCCAACGGGCCCTTCTCTTTCTATGGAGATTATCAGAGCGAGTATTATGGCCCTGGAAGCAATTATGATTTC

CTCGAGTAGGGGTTGCCCGGGAAGAGAAAGATACCTCTAATAGTCTCGCTCATAATACCGGGACCTTCGTTAATACTAAAGAARGGNGTYCCNGGNCC

### Eo Brachyury: Accession number EU741247

V P T N E M N V T K N G R R M F P V L K V S V S G L D P N A M Y S F L L D F V A A D N H R W K Y

GGNCCNACNAAYGARATGAACGTGACCAAGAACGGCAGGCGGATGTTCCCCGTGCTGAAGGTGAGCGTGTCCGGCCTGGACCCCAACGCTATGTACTCCTTCCTGCTGGACTTCGTGGCCGCCGACAATCACCGCTGGAAGTAC

TTGCACTGGTTCTTGCCGTCCGCCTACAAGGGGCACGACTTCCACTCGCACAGGCCGGACCTGGGGTTGCGATACATGAGGAAGGACGACCTGAAGCACCGGCGGCTGTTAGTGGCGACCTTCATG

V N G E W V P G G K P E P Q A P S C V Y I H P D S P N F G A H W M K A P V S F S K V K L T N K L

GTGAACGGGGAGTGGGTCCCCGGGGGCAAACCCGAGCCGCAGGCCCCGAGCTGCGTCTACATCCACCCAGACTCGCCCAACTTCGGAGCGCACTGGATGAAGGCGCCCGTTTCCTTCAGCAAAGTCAAACTCACCAACAAGCTC

CACTTGCCCCTCACCCAGGGGCCCCCGTTTGGGCTCGGCGTCCGGGGCTCGACGCAGATGTAGGTGGGTCTGAGCGGGTTGAAGCCTCGCGTGACCTACTTCCGCGGGCAAAGGAAGTCGTTTCAGTTTGAGTGGTTGTTCGAG

N G G G Q I M L N S L H K Y E P R I H I V R V G G P Q R M I T S H S F P E T Q F I A V T A Y Q N

AATGGAGGGGGGCAGATCATGTTGAACTCCTTGCACAAGTATGAGCCTAGGATTCATATAGTGAGAGTTGGTGGCCCTCAGCGTATGATAACCAGCCATTCCTTCCCAGAGACCCAGTTTATAGCTGTGACAGCTTATCAGAAT

TTACCTCCCCCCGTCTAGTACAACTTGAGGAACGTGTTCATACTCGGATCCTAAGTATATCACTCTCAACCACCGGGAGTCGCATACTATTGGTCGGTAAGGAAGGGTCTCTGGGTCAAATATCGACACTGTCGAATAGTCTTA

E E I T A L K I K Y N P F A K A

GAGGAGATCACAGCTTTAAAAATTAAATAC

CTCCTCTAGTGTCGAAATTTTTAATTTATGTTRGGNAARCGNTTYGC

## **Eo Otx2: Accession number EU741248**

N G G Q S K V R P A K K K S S P A R E V S S E S G T S G Q F T P P S S T S V P A I S S S S A P V

AACGGGGGCCAGAGCAAAGTGAGGCCGGCCAAGAAGAAGAGCTCCCCGGCTCGCGAAGTAAGTTCGGAGAGCGGCACCAGCGGGCAGTTCACCCCCCCCTCCAGCACCTCCGTCCCAGCCATTTCCAGCAGCAGTGCCCCCGTG

TCACTCCGGCCGGTTCTTCTTCTCGAGGGGCCGAGCGCTTCATTCAAGCCTCTCGCCGTGGTCGCCCGTCAAGTGGGGGGGGAGGTCGTGGAGGCAGGGTCGGTAAAGGTCGTCGTCACGGGGGCAC

S I W S P A S I

TCTATC

AGATAGACCTCRGGNCGNAGNTA

#### Eo Otx5 : Accession number AF518735

Y P D I F M R E E V A L K I N L P E S R V Q V W F K N R R A K C R Q Q Q Q Q S S G Q A K A R P A

TAYCCNGAYATNTTYATGCGGGAGGAGGTAGCGCTCAAGATCAACCTGCCGGAGTCCCGGGTGCAGGTTTGGTTCAAGAACCGACGGGCCAAGTGCCGCCAGCAGCAGCAGCAGAGCAGTGGCCAGGCCAAGGCGCGCCCGGCC

GCCCTCCTCCATCGCGAGTTCTAGTTGGACGGCCTCAGGGCCCACGTCCAAACCAAGTTCTTGGCTGCCCGGTTCACGGCGGTCGTCGTCGTCGTCTCGTCACCGGTCCGGTTCCGCGCGGGCCGG

K K K S S P A R E T S S E T S T N G Q Y S P P P A G T S G T P S S T A S A T V S I W S P A S I S

AAGAAGAAGAGCTCGCCGGCCCGGGAGACCAGCTCGGAAACCAGCACCAACGGGCAATACAGCCCCCCGCCCGCCGGCACCTCGGGCACCCCCAGCTCCACGGCCAGCGCCACCGTCTCCATCTGGAGCCCGGCCTCCATCTCC

TTCTTCTTCTCGAGCGGCCGGGCCCTCTGGTCGAGCCTTTGGTCGTGGTTGCCCGTTATGTCGGGGGGCGGGCGGCCGTGGAGCCCGTGGGGGTCGAGGTGCCGGTCGCGGTGGCAGAGGTAGACCTCGGGCCGGAGGTAGAGG

P I P D P L A S S A T P C M Q R S A A Y P M T Y T Q G A G Y P Q S Y A G S S S Y F T G L D C G S

CCCATCCCCGACCCCCTGGCCTCCTCGGCCACCCCCTGCATGCAGCGCTCTGCCGCCTACCCGATGACCTACACCCAGGGCGCCGGCTACCCCCAGAGCTACGCCGGCTCCTCCTCCTACTTCACCGGCCTGGACTGCGGCTCC

GGGTAGGGGCTGGGGGACCGGAGGAGCCGGTGGGGGACGTACGTCGCGAGACGGCGGATGGGCTACTGGATGTGGGTCCCGCGGCCGATGGGGGTCTCGATGCGGCCGAGGAGGAGGATGAAGTGGCCGGACCTGACGCCGAGG

Y L S P M H P Q L S A P G A A L S P I A A P A M G T H L S Q S P A S L S S Q G F G T A G L S F G

TACCTCTCGCCCATGCACCCGCAGCTCTCCGCCCCCGGCGCCGCCCTGAGCCCCATCGCCGCCCCGGCCATGGGCACCCACCTCAGCCAGTCCCCGGCCTCCCTGTCCAGCCAGGGCTTCGGCACGGCCGGCCTGAGCTTTGGC

ATGGAGAGCGGGTACGTGGGCGTCGAGAGGCGGGGGCCGCGGCGGGACTCGGGGTAGCGGCGGGGCCGGTACCCGTGGGTGGAGTCGGTCAGGGGCCGGAGGGACAGGTCGGTCCCGAAGCCGTGCCGGCCGGACTCGAAACCG

S V D C L D Y K D Q T A S W K L N F N A T D C L D Y K D Q S

TCGGTGGATTGCTTAGACTACAAGGATCAGACGGCCTCTTGGAAACTCAACTTCAACGCCACGGACTGCTTG

AGCCACCTAACGAATCTGATGTTCCTAGTCTGCCGGAGAACCTTTGAGTTGAAGTTGCGGTGCCTGACGAACCTRATRTTYCTRGTYTC

**Figure S1: Partial *E. orbicularis Lim1*, *Brachyury*, *Otx2* and *Otx5* sequences used as probes in this study.**

##### Partial sequences with their deduced amino acid sequences are indicated for each gene. The sequence of the degenerate primers used in the PCR amplification is shown shaded in grey, with an arrow pointing to their 3’ end.
